# The prognostic value of N-terminal proB-type natriuretic peptide in patients with acute respiratory distress syndrome

**DOI:** 10.1038/srep44784

**Published:** 2017-03-21

**Authors:** Chih-Cheng Lai, Mei-I. Sung, Chung-Han Ho, Hsiao-Hua Liu, Chin-Ming Chen, Shyh-Ren Chiang, Chien-Ming Chao, Wei-Lun Liu, Shu-Chen Hsing, Kuo-Chen Cheng

**Affiliations:** 1 Department of Intensive Care Medicine, Chi Mei Medical Center, Liouying, Taiwan; 2 Departments of Internal Medicine, Chi Mei Medical Center, Tainan, Taiwan; 3 Departments of Medical Research, Chi Mei Medical Center, Tainan, Taiwan; 4 Chia Nan University of Pharmacy & Science, Tainan, Taiwan; 5 Departments of Intensive Care Medicine, Chi Mei Medical Center, Tainan, Taiwan; 6 Department of Safety Health and Environmental Engineering, Chung Hwa University of Medical Technology, Tainan, Taiwan

## Abstract

We investigated whether N-terminal proB-type natriuretic peptide (NT-proBNP) predicts the prognosis of patients with acute respiratory distress syndrome (ARDS). Between December 1, 2012, and May 31, 2015, this observational study recruited patients admitted to our tertiary medical center who met the Berlin criteria for ARDS and who had their NT-proBNP measured. The main outcome was 28-day mortality. We enrolled 61 patients who met the Berlin criteria for ARDS: 7 were classified as mild, 29 as moderate, and 25 as severe. The median APACHE II scores were 23 (interquartile range [IQR], 18–28), and SOFA scores were 11 (IQR, 8–13). The median lung injury score was 3.0 (IQR, 2.50–3.25), and the median level of NT-proBNP was 2011 pg/ml (IQR, 579–7216). Thirty-four patients died during this study, and the 28-day mortality rate was 55.7%. Patients who die were older and had significantly (all p < 0.05) higher APACHE II scores and NT-proBNP levels than did patients who survived. Multivariate analysis identified age (HR: 1.546, 95% CI: 1.174–2.035, p = 0.0019) and NT-proBNP (HR: 1.009, 95% CI: 1.004–1.013, p = 0.0001) as significant risk factors of death. NT-proBNP was associated with poor outcomes for patients with ARDS, and its level predicted mortality.

Acute respiratory distress syndrome (ARDS) can develop as a consequence of various direct pulmonary insults, e.g., infectious pneumonia, aspiration, traumatic lung contusion, or of indirect lung injuries, e.g., sepsis, shock, massive blood transfusion, and non-pulmonary trauma^1^. Critically ill patients with ARDS usually require mechanical ventilation. The American and European Consensus Conference (AECC)^2^ defined ARDS and acute lung injury (ALI) as: (1) acute onset of respiratory distress, (2) bilateral infiltrates on chest radiography, (3) the absence of cardiogenic pulmonary edema, and (4) severe hypoxemia. However, AECC criteria do not reflect the severity or outcomes of ARDS^3^,^4^. Therefore, in 2012, the Berlin definition^5^,^6^ proposed three categories of ARDS based of the degree of hypoxemia: mild (200 mmHg < PaO_2_/FiO_2_ ≤ 300 mmHg), moderate (100 mmHg < PaO_2_/FiO_2_ ≤ 200 mmHg), and severe (PaO_2_/FiO_2_ ≤ 100 mmHg) with a positive end expiratory pressure (PEEP) ≥ 5 cm H_2_O. Although the Berlin criteria better predictors of mortality in patients with ARDS than is the AECC definition, the absolute value of the area under the receiver-operating curve (ROC) for predicting risk of death was only 0.577^7^. All of these findings should indicate that the initial hypoxemic level is not an optimal predictor for an outcome prognosis in patients with ARDS, and that we need other significant prognostic factors for predicting the outcomes.

B-type natriuretic peptide (BNP) was first described in the porcine brain^8^, but BNP in humans originate primarily from the heart’s ventricular myocardium^9^. The secretion of BNP is mediated by the ventricles of the heart in response to excessive stretching of heart muscle cells^10^. BNP is synthesized as a prehormone (proBNP) comprising 108 amino acids, and it is cleaved into the biologically active 32-amino acid BNP, which represents the C-terminal fragment, and the biologically inactive 76-amino acid N-terminal fragment (NT-proBNP)^10^. Both BNP and NT-proBNP are diagnostic tools for assessing fluid status and cardiac strain in congestive heart failure, pulmonary hypertension, and other cardiovascular diseases. In addition to their diagnostic usefulness, BNP and NT-proBNP can provide strong prognostic information of an unfavorable outcome in patients with heart failure^10^,^11^. Recently, several studies^12^,^13^,^14^,^15^,^16^,^17^,^18^,^19^ have reported that BNP or NT-proBNP was elevated in patients with ARDS. However, only Determann *et al*.^12^ and Park *et al*.^13^,^14^ focused on NT-proBNP; all of the others^15^,^16^,^17^,^18^,^19^ investigated the diagnostic or prognostic role of BNP in patients with ARDS. Thus, the usefulness of NT-proBNP predicting outcomes of patients with ARDS remains unclear. Therefore, we investigated whether the level of NT-proBNP is a precise predictor of the prognosis in patients with ARDS.

## Patients and Methods

### Patients and Hospital Setting

This study was done at Chi Mei Medical Center, a 1288-bed tertiary medical center with 96 intensive care unit (ICU) beds for adults: 57 medical and 39 surgical. The care in the ICU is covered by intensivists, senior residents, nurses, respiratory therapists, dietitians, physical therapists, and clinical pharmacists. The ICU team makes rounds at least once daily, and respiratory therapists are responsible for managing all mechanical ventilation (MV), including weaning and spontaneous breathing trials (SBT). Patients admitted to ICUs were screened daily to identify who met the Berlin definition of ARDS, as previously described^20^, and were recruited between December 2012 and May 2015. Exclusion criteria were a cardiac echo showing a left ventricle ejection fraction ≤50%, cardiopulmonary cerebral resuscitation just before enrollment, and inconsistent findings of ARDS on chest radiography. Data were collected on a routine basis, and the analysis was conducted retrospectively. The study was approved by the institutional review board of Chi Mei Medical Center, and informed consent was waived. All methods were performed in accordance with the relevant guidelines and regulations

### Measuring Variables

The following information of the enrolled patients was prospectively recorded: age, gender, cause of ARDS, clinical features, laboratory data, comorbidities (congestive heart failure, chronic lung diseases, end-stage renal disease, liver cirrhosis, diabetes mellitus, acute or chronic encephalopathy, cancer, and an immunocompromised condition), organ dysfunction, Acute Physiology and Chronic Health Evaluation (APACHEII) score^21^, and Lung Injury Score during the first 24 hours in the ICU before and after using standard MV.

The primary outcome was 28-day mortality regardless of cause. In addition, arterial blood gas (ABG) and ventilator settings on the enrolment day (Day 0) were recorded. Mandatory standard MV settings were used: Mode = volume assist/control; Tidal Volume (V_T_) = 7 ml/kg of predicted body weight (PBW); Ventilator Rate = sufficient to maintain 35–50 mmHg of PaCO_2_; plus the PEEP and FiO_2_ settings previously described^22^. After the MV settings had been made, patients were left undisturbed and blood gases were collected 30 minutes later. Throughout the acute course of MV care, V_T_ was set at 5–9 ml/kg PBW with a plateau pressure <30 cm H_2_O and a ventilation rate that would maintain PaCO_2_ at 35–50 mmHg and PEEP at ≥5 cm H_2_O. Serum levels of NT-proBNP were measured using an electrochemiluminescence immunoassay (Roche Diagnostics Nederland BV, Almere, the Netherlands) in the morning at the first day of enrollment.

### Definitions

As previously stated^20^,^23^,^24^, shock was defined as systolic blood pressure (SBP) of ≤90 mmHg or a mean arterial pressure (MAP) ≤ 65 mmHg for at least 1 hour despite adequate fluid resuscitation; or the need for vasoactive agents (dopamine ≥5 mg/kg/min) to maintain SBP ≥90 mmHg or MAP ≥65 mmHg. Metabolic failure was defined as pH ≤ 7.30 or a base deficit ≥5.0 mEq/L and a plasma lactate level >3 mmol/L. Hematologic failure was defined as a platelet count <80,000/mm^3^, or a 50% decrease in platelet count from the highest value recorded over the previous 3 days. Kidney failure was defined as oliguria with an average urine output <0.5 mL/kg/h for 4 hours despite adequate fluid resuscitation, or creatinine ≥2 mg/dL. Hepatic failure was defined as a markedly increased serum bilirubin level ≥4 mg/dL.

### Statistical Analysis

Continuous variables were reported as medians with interquartile range (IQR), and categorical variables were presented as frequency counts and percentage. The differences in baseline characteristics and clinical variables between the Survival and Mortality groups were evaluated using Wilcoxon rank-sum test for continuous variables and Pearson’s χ^2^ test for categorical variables. Significance was set at p < 0.05 (two-tailed).

Cox proportional hazard regression analyses were used to estimate the risk of time-to-mortality, death at Day 28, and to present the possible predictors of mortality. Data are presented as a hazard ratio (HR) plus a 95% confidence interval (CI). The association between all potential predictors and mortality were evaluated using univariate and multivariate analyses. For the multivariate analysis, all potential predictors were considered, and the forward stepwise selection approach was used for the final evaluation with the significance criterion of 0.20 for covariate entry and 0.10 for covariate removal. The validity assumptions of the Cox proportional hazard regression were assessed using Schoenfeld residuals for all variables. In addition, for assessing the accuracy of NT-proBNP, the ROC curve analysis was used. SAS 9.4 for Windows (SAS Institute, Cary, NC, USA) was used for all analyses. The ROC curves was estimated using Stata 12 (Stata Corp., College Station, TX, USA).

## Results

**Figure 1:**
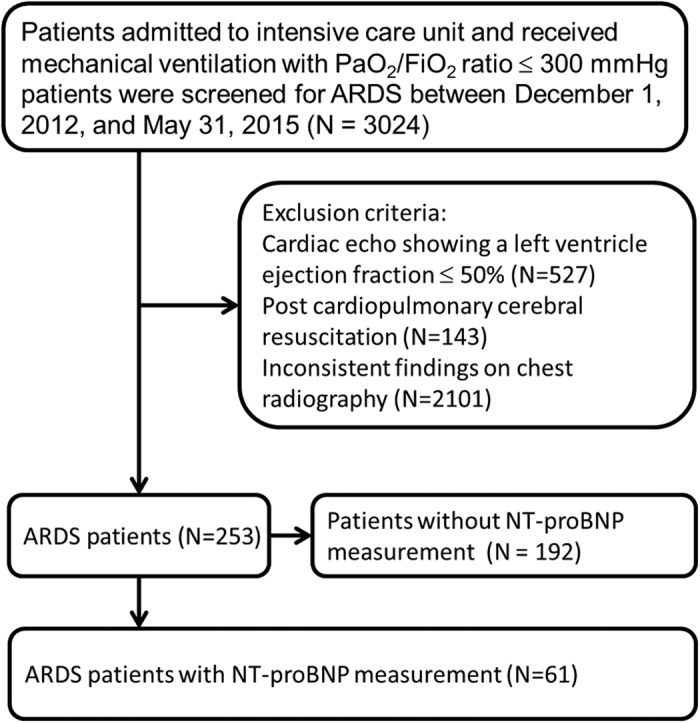
The algorithm of patient enrollment.

During this 30-month study, 61 patients (median age: 65 years, IQR, 56.0–78.5 years; men: 39 [63.9%]) who met the Berlin criteria for ARDS and had an initial measurement of NT-proBNP were enrolled (Fig. 1 and Table 1). Most of these patients had been transferred from the General Ward (23 [37.7%]) and the Emergency Department (21 [34.4%]). Forty-nine (80%) patients were admitted from medical departments. Pneumonia (55 [90.2%]) and nonpulmonary sepsis (6 [9.8%]) were the etiologies of ARDS. APACHE II scores were 23 (IQR, 18–28), and SOFA scores were 11 (IQR, 8–13). The lung injury score was 3.0 (IQR, 2.50–3.25), and the baseline PaO_2_/FiO_2_ was 114.7 mmHg (IQR, 76.0–166.8 mmHg). The level of NT-proBNP was 2011 pg/ml (IQR, 579–7216 pg/ml). Based on the Berlin criteria, 29 patients had moderate ARDS (47.5%), 25 had severe ARDS (41.0%), and 7 had mild ARDS (11.5%). Shock was the most common systemic failure, followed by renal, metabolic, and hematologic failure. The mean length of ICU stay was 16 days and of hospital stays was 20 days. Table 2 showed that the level of NT-proBNP was not correlated with the severity of ARDS.

**Figure 2:**
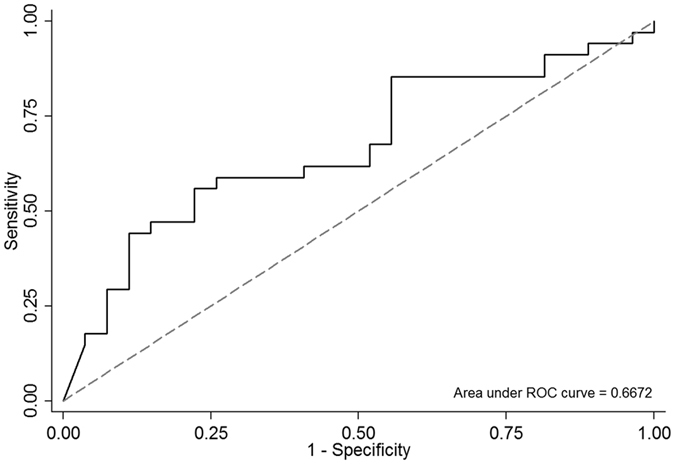
Receiver operating characteristic curve of NT-proBNP for predicting the mortality in ARDS patients.

Thirty-four patients died during this study, and the 28-day mortality rate was 55.7%. Patients who died were older and had higher APACHE II scores and NT-proBNP levels than did patients who survived (all p < 0.05) (Table 3). The accuracy area under the curve (AUC) of NT-proBNP for predicting the mortality of ARDS patients was 0.667 (95% CI: 0.529–0.805) (Fig. 2). In contrast, gender, SOFA scores, lung injury scores, blood pressure, body mass index, V_T_/PBW, baseline data of arterial blood gas, number of comorbidities, oxygenation, and the PaO_2_/FiO_2_ ratio on Day 0, were not significantly different. Finally, survivors had longer ICU and hospital stays than did patients who died.

A multivariate Cox proportional hazard regression analysis showed that age (HR: 1.546, 95% CI: 1.174–2.035), APACHE II scores (HR: 1.627, 95% CI: 0.992–2.670), and NT-proBNP levels (HR: 1.009, 95% CI: 1.004–1.013) were significant risk factors for mortality (Table 4).

## Discussion

We had several significant findings. First, the blood NT-proBNP level was significantly associated with the prognosis of ARDS patients. Patients who died had significantly higher NT-proBNP levels than did survivors (3956 pg/mL vs. 1190 pg/mL; p = 0.026). The adjusted HR for mortality was 1.009 (95% CI: 1.004–1.013; p < 0.0001), a finding consistent with other BNP studies. Sun *et al*.^19^ reported that, of 59 patients with ALI/ARDS, BNP levels were significantly higher in the nonsurvivor group than in the survivor group (267 pg/mL vs. 128 pg/mL; p < 0.01). Karmpaliotis *et al*.^15^, in a study of 50 ARDS patients, found a strong graded relationship with mortality risk. Lin *et al*.^16^ reported that BNP levels differed significantly between the 31 survivors and 55 nonsurvivors [(179.5 +/− 84.5) ng/L vs. (550.8 +/− 337.1) ng/L; p < 0.05]. However, in contrast to most of the above studies that used the AECC criteria to diagnose ARDS, and BNP to assess outcomes, our study used the Berlin criteria to diagnose ARDS and NT-proBNP to assess outcomes. Overall, these findings should suggest the clinical usefulness of NT-proBNP and BNP for assessing the prognosis of ARDS patients. Both NT-proBNP and BNP are significantly associated with worse outcomes for ARDS patients.

Second, although our enrolled ARDS patients had undergone a cardiac echo to exclude possible cardiogenic pulmonary edema (CPE), all had elevated NT-proBNP levels (range: 579–7216 pg/mL). They also tended to have increasing NT-proBNP levels and ARDS severity. This finding is in line with other reports^15^,^16^ about the diagnostic utility of BNP in ARDS patients. The mechanism might be myocardial injury because of hypoxia and the release of inflammatory mediators. In addition, pulmonary dysfunction and increasing pulmonary pressure in ARDS can increase BNP synthesis and release. Another possible explanation might be that it is difficult to totally exclude CPE, even when using cardiac echo. Therefore, NT-proBNP and BNP can be elevated in ARDS patients. However, we did not try to find the cutoff value of NT-proBNP level to help differentiate diagnosis between ARDS and CPE. Additional investigation is warranted to evaluate the diagnostic performance of NT-proBNP for discriminating between ARDS and CPE.

Third, we found that the severity of ARDS based on the Berlin criteria was not significantly associated with mortality. Although mortality significantly increased with the severity of ARDS in the original study on the Berlin criteria for ARDS^5^, the findings of Hernu *et al*.^25^ were inconsistent: 28-day mortality rates were 30.9% for mild, 27.9% for moderate, and 49.3% for severe ARDS. In our study, mortality rates between mild (48.0%), moderate (48.0%), and severe (48.0%) ARDS were not significantly different, nor were the mortality rates significantly associated with the initial ARDS stage in a multivariate analysis. These findings might indicate that current Berlin criteria for ARDS are not an accurate predictor of mortality^20^,^25^.

This study had some limitations. First, it was done in a single medical center, and the number of patients was limited. Our findings might not be generalizable to other hospitals. Second, we followed only a lung-protective strategy, but did not provide a specific protocol for adjusting the setting of mechanical ventilator settings throughout the ICU stay. However, our study design truly reflects the situation in the real world. Third, some management methods for ARDS, such as prone position, high frequency oscillatory ventilation, and the lung recruitment maneuver, were not recorded.

## Conclusion

We conclude that NT-proBNP is a good predictor of outcomes in patients with ARDS. In contrast, the current Berlin criteria of ARDS severity cannot accurately predict outcomes.

## Additional Information

**How to cite this article:** Lai, C.-C. *et al*. The prognostic value of N-terminal proB-type natriuretic peptide in patients with acute respiratory distress syndrome. *Sci. Rep.* **7**, 44784; doi: 10.1038/srep44784 (2017).

**Publisher's note:** Springer Nature remains neutral with regard to jurisdictional claims in published maps and institutional affiliations.

## Figures and Tables

**Table 1:** Demographics of patients.

Characteristic	Total (n = 61)	Survivors (n = 27)	Non-survivors (n = 34)
Males, n (%)	39 (63.9)	18 (66.7)	21 (61.8)
Age, years	65 (56.0–78.5)	57 (46–67)	73 (64–81)
Source			
Emergency Department, n (%)	21 (34.4)	12 (44.4)	9 (26.5)
General Ward, n (%)	23 (37.7)	7 (25.9)	16 (47.1)
Other hospital, n (%)	17 (27.9)	8 (29.6)	9 (26.5)
Department			
Medicine, n (%)	49 (80.3)	23 (85.2)	26 (76.5)
Surgery, n (%)	12 (19.7)	4 (14.8)	8 (23.5)
Cause of ARDS			
Pneumonia, n (%)	55 (90.2)	24 (88.9)	31 (91.2)
Sepsis other than lung infection, n (%)	6 (9.8)	3 (11.1)	3 (8.8)
APACHE II score	23 (18–28)	23 (16–24)	26 (21–33)
SOFA score	11 (8–13)	10 (7–13)	11 (9–13)
Lung injury score	3.0 (2.50–3.25)	3.0 (2.50–3.25)	2.88 (2.50–3.25)
V_T_, ml/kg PBW	7.4 (6.7–8.0)	7.5 (6.7–8.4)	7.3 (6.8–7.9)
Arterial blood gas (Day 0)			
pH	7.39 (7.34–.44)	7.41 (7.36–7.45)	7.37 (7.32–7.43)
PaCO_2,_ cm H_2_O	34.0 (28.6–39.7)	31.6 (29.2–38.9)	34.2 (28.4–42.7)
PaO_2,_ cm H_2_O	81.0 (65.3–114.6)	81.7 (65.4–131.8)	79.2 (63.4–107.5)
HCO3, mEq/l	20.6 (18.3–23.7)	20.9 (18.3–26.0)	20.1 (17.5–23.6)
FiO_2_	0.95 (0.65–1.0)	0.8 (0.7–1.0)	1.0 (0.65–1.0)
PaO_2_/FiO_2_ mmHg	114.7 (76.0–166.8)	111.8 (73.6–172.3)	116.5 (78.4–151.5)
NT-proBNP, pg/ml	2011 (579–7216)	1190 (398–3530)	3956 (771–9838)
Oxygenation based on Berlin criteria			
Mild ARDS, n (%)	7 (11.5)	1 (3.7)	6 (17.6)
Moderate ARDS, n (%)	29 (47.5)	13 (48.1)	16 (47.1)
Severe ARDS, n (%)	25 (41.0)	13 (48.1)	12 (35.3)
Organ dysfunction			
Cardiovascular, n (%)	46 (75.4)	19 (70.4)	27 (79.4)
Kidney, n (%)	21 (34.4)	5 (18.5)	16 (47.1)
Metabolic, n (%)	15 (24.6)	5 (18.5)	10 (29.4)
Hematologic, n (%)	14 (23.0)	7 (25.9)	7 (20.6)
Hepatic, n (%)	2 (3.3)	1 (3.7)	1 (2.9)
Number of comorbidities	1.0 (1.0–2.0)	1 (0–2)	1 (1–2)
Number of organ dysfunctions	1.0 (1.0–2.0)	1 (1–2)	2 (1–3)
Length of ICU stay, days	16.0 (10.0–21.0)	19 (13–35)	13 (10–17)
Length of hospital stay, days	20.0 (13.5–35.0)	35 (28–50)	15 (10–20)

Values are median (IQR) unless otherwise indicated.

**Table 2:** Relationship of oxygenation (based on Berlin criteria) and NT-proBNP

PaO_2_/FiO_2_ ratio	Mild ARDS (n = 7)	Moderate ARDS (n = 29)	Severe ARDS (n = 25)	*p*
NT-proBNP	7695 (4755–8456)	1202 (565–6692)	1938 (557–3789)	0.114

**Table 3:** Comparison of patients with 28-day survival and mortality.

Characteristic	Mortality (n = 34, 55.7%)	Survival (n = 27, 44.3%)	*p value*
Age	73 (64–81)	57 (46–67)	<**0**.**001**
Gender			0.791
Male	21 (61.8%)	18 (66.7%)	
Female	13 (38.2%)	9 (33.3%)	
APACHE II score	26 (21–33)	23 (16–24)	**0**.**008**
SOFA score	11 (9–13)	10 (7–13)	0.221
Acute lung injury score	2.88 (2.50–3.25)	3.0 (2.50–3.25)	0.540
Mean arterial pressure	69.2 (61.0–79.7)	71.7 (66.6–86.0)	0.257
BMI	22.7 (19.8–25.0)	24.2 (21.6–27.6)	0.235
V_T_, ml/kg PBW	7.3 (6.8–7.9)	7.5 (6.7–8.4)	0.532
PaO_2_/FiO_2_, mmHg	116.5 (78.4–151.5)	111.8 (73.6–172.3)	0.738
pH	7.374 (7.324–7.435)	7.416 (7.366–7.454)	0.117
NT-proBNP, pg/ml	3956 (771–9838)	1190 (398–3530)	**0**.**026**
Number of comorbidities	1 (1–2)	1 (0–2)	0.598
Number of organ dysfunctions	2 (1–3)	1 (1–2)	0.204
Length of ICU stay, days	13 (10–17)	19 (13–35)	<0.001
Length of hospital stay, days	15 (10–20)	35 (28–50)	<0.001

**Table 4:** Predictors of outcome for 28-day non-survivors.

Characteristic	Univariate HR (95% CI)	*p value*	Multivariate HR (95% CI)	*p value*
Age (per 10)	1.582 (1.227–2.041)	<0.001	1.546 (1.174–2.035)	0.0019
APACHE II score (per 10)	1.839 (1.155–2.928)	0.010	1.627 (0.992–2.670)	0.0539
SOFA score (per 5)	1.311 (0.807–2.131)	0.274		
Acute lung injury score	0.725 (0.334–1.574)	0.415		
Mean arterial pressure	0.987 (0.967–1.007)	0.213		
BMI	0.955 (0.885–1.031)	0.237		
V_T_, ml/kg PBW	0.885 (0.665–1.176)	0.399		
PaO_2_/FiO_2_, (mmHg)	1.001 (0.995–1.007)	0.655		
pH value (per 0.1)	0.788 (0.479–1.296)	0.347		
NT-proBNP (per 100)	1.006 (1.002–1.010)	0.002	1.009 (1.004–1.013)	0.0001
Number of comorbidities	0.983 (0.661–1.463)	0.934		
Number of organ dysfunctions	1.302 (0.950–1.785)	0.101		
